# The adaptation of bumblebees to extremely high elevation associated with their gut microbiota

**DOI:** 10.1128/msystems.01219-23

**Published:** 2024-02-08

**Authors:** Jiu-Hong Dong, Xin Xu, Zong-Xin Ren, Yan-Hui Zhao, Yaran Zhang, Li Chen, You Wu, Guotao Chen, Ruiqing Cao, Qi Wu, Hong Wang

**Affiliations:** 1 State Key Laboratory of Mycology, Institute of Microbiology, Chinese Academy of Sciences, Beijing, China; 2 CAS Key Laboratory for Plant Diversity and Biogeography of East Asia, Kunming Institute of Botany, Chinese Academy of Sciences, Kunming, China; 3 University of Chinese Academy of Sciences, Beijing, China; Pacific Northwest National Laboratory Biological Sciences Division, Richland, USA

**Keywords:** high elevation, adaptive selection, *Bombus*, bumblebee microbiome, gut microbiome, metagenome, population structure

## Abstract

Bumblebees are among the most abundant and important pollinators for sub-alpine and alpine flowering plant species in the Northern Hemisphere, but little is known about their adaptations to high elevations. In this article, we focused on two bumblebee species, *Bombus friseanus* and *Bombus prshewalskyi*, and their respective gut microbiota. The two species, distributed through the Hengduan Mountains of southwestern China, show species replacement at different elevations. We performed genome sequencing based on 20 worker bee samples of each species. Applying evolutionary population genetics and metagenomic approaches, we detected genes under selection and analyzed functional pathways between bumblebees and their gut microbes. We found clear genetic differentiation between the two host species and significant differences in their microbiota. Species replacement occurred in both hosts and their bacteria (*Snodgrassella*) with an increase in elevation. These extremely high-elevation bumblebees show evidence of positive selection related to diverse biological processes. Positively selected genes involved in host immune systems probably contributed to gut microbiota changes, while the butyrate generated by gut microbiota may influence both host energy metabolism and immune systems. This suggests a close association between the genomes of the host species and their microbiomes based on some degree of natural selection.

**IMPORTANCE:**

Two closely related and dominant bumblebee species, distributed at different elevations through the Hengduan Mountains of southwestern China, showed a clear genomic signature of adaptation to elevation at the molecular level and significant differences in their respective microbiota. Species replacement occurred in both hosts and their bacteria (*Snodgrassella*) with an increase in elevation. Bumblebees’ adaptations to higher elevations are closely associated with their gut microbiota through two biological processes: energy metabolism and immune response. Information allowing us to understand the adaptive mechanisms of species to extreme conditions is implicit if we are to conserve them as their environments change.

## INTRODUCTION

Environments at high elevations are characterized by low temperature, high radiation, and hypoxia, presenting strong selective pressures on their resident fauna (1). Genetic variants, contributing to animals’ adaptations to high elevations, have been found to be present in Tibetans (2), yaks (3), Tibetan pigs (4), and some insects including honeybees (5) and locusts (6). While bumblebees (Apidae: *Bombus*) remain one of the most important pollinators of wildflowers and crops, few studies focus on their adaptation to high elevations. The most recent research work provides a comparative study focusing on the genomes of 17 *Bombus* species within 15 subgenera related to high-elevation adaptation (7). However, the authors found no positive selection genes in common within four high-elevation species. This can be attributed to multiple factors. First, each bumblebee species may have a different genetic architecture of adaptation. Second, bumblebees’ adaptation to high elevations may be a polygenic trait, with different loci selected in each species. Besides, phylogenetic analyses on multispecies divergence data identify long-term genetic variations that have been fixed in each species, which may lead to missed detection of recent adaptive signals. Therefore, to understand bumblebees’ high-elevation adaptation strategies, more species need to be investigated using more sensitive methods. Comparatively investigating closely related species that occupy different elevation ranges based on population approaches may be useful in identifying more candidate genes under adaptive selection (8, 9).

Accumulating evidence indicates that gut microbes regulate their host’s physiology and health. In particular, specific gut microbes are now associated with high-elevation adaptations, including the people of Tibet (10, 11) and some of their domesticated mammals (12, 13). However, the composition of the gut microbiota of eusocial honeybees and bumblebees appears simpler compared to that of mammals (14, 15). Just a handful of taxa of bacteria dominate the bumblebee gut floras, including *Snodgrassella*, *Gilliamella*, *Schmidhempelia*, *Bifidobacteriaceae,* and two clusters within the *Lactobacillaceae*. Previous studies showed that these bacteria exhibited host specificity, substantial strain-level diversity, differences in genome structure and function, and spatial niche partitioning within individual hosts (15–17). Therefore, it is reasonable to assume that the gut microbiota of high-elevation bumblebees may possess a unique composition and function that may be indicative of their adaptation to certain extreme conditions.

The Hengduan Mountains of southwestern China are classified as a global hotspot (18) and one of several centers of bumblebee diversity. In general, bumblebees remain the most abundant and important pollinator in the Order Hymenoptera for sub-alpine and alpine plant species (19, 20). As the elevation zones increase from 2,000 to 4,000 m, closely related bumblebee species are replaced (20). The most notable replacement occurs between two species in subgenus *Melanobombus*: *Bombus friseanus* and *Bombus prshewalskyi*. These two species shared a common ancestor 4.5 million years ago (18). At these elevations, *B. friseanus* is distributed from 1,500 to 3,500 m, while *B. prshewalskyi* is found above the tree line from 3,500 to 5,000 m.

*B. friseanus* is endemic to the Hengduan Mountains, foraging in subalpine meadows bordering conifer forests, and is abundant between 2,700 and 3,300 m on the Yulong Snow Mountain (20). At 3,200 m, *B. friseanus* accounted for 68% of all bumblebee species *in situ*, dominating bumblebee–plant networks (19). In contrast, *B. prshewalskyi* is distributed from the Hengduan Mountain westward through the Tibetan Plateau and the Eastern Himalayas (18). It is abundant above 4,200 m on the Baima Snow Mountain. Species replacements along elevation gradients are common in montane sites (21, 22), suggesting different adaptations to localized biotic and abiotic factors among closely related species (23). Therefore, we propose that *B. friseanus* and *B. prshewalskyi* provide a representative model for understanding the adaptation mechanism of wild bumblebees to extremely high elevations. We hypothesize that, compared to *B. friseanus*, *B. prshewalskyi* may have unique characteristics in its genome and gut microbiota, allowing this insect to survive at extremely high elevations.

We collected workers of *B. friseanus* and *B. prshewalskyi* and extracted their guts. Deep-coverage metagenomic shotgun sequencing was performed for host and gut microbiomes. We conducted evolutionary population genetic analyses to identify genetic variants related to bumblebee adaptations to extremely high elevations. Comparative metagenomic analyses were applied to provide an overview of the gut microbiota diversity and functional differences. Integration of these results should deepen our understanding of ecophysiological strategies employed by bumblebees to survive in extreme environmental conditions.

## RESULTS

### Metagenomic sequencing of bumblebees

**Fig 1:**
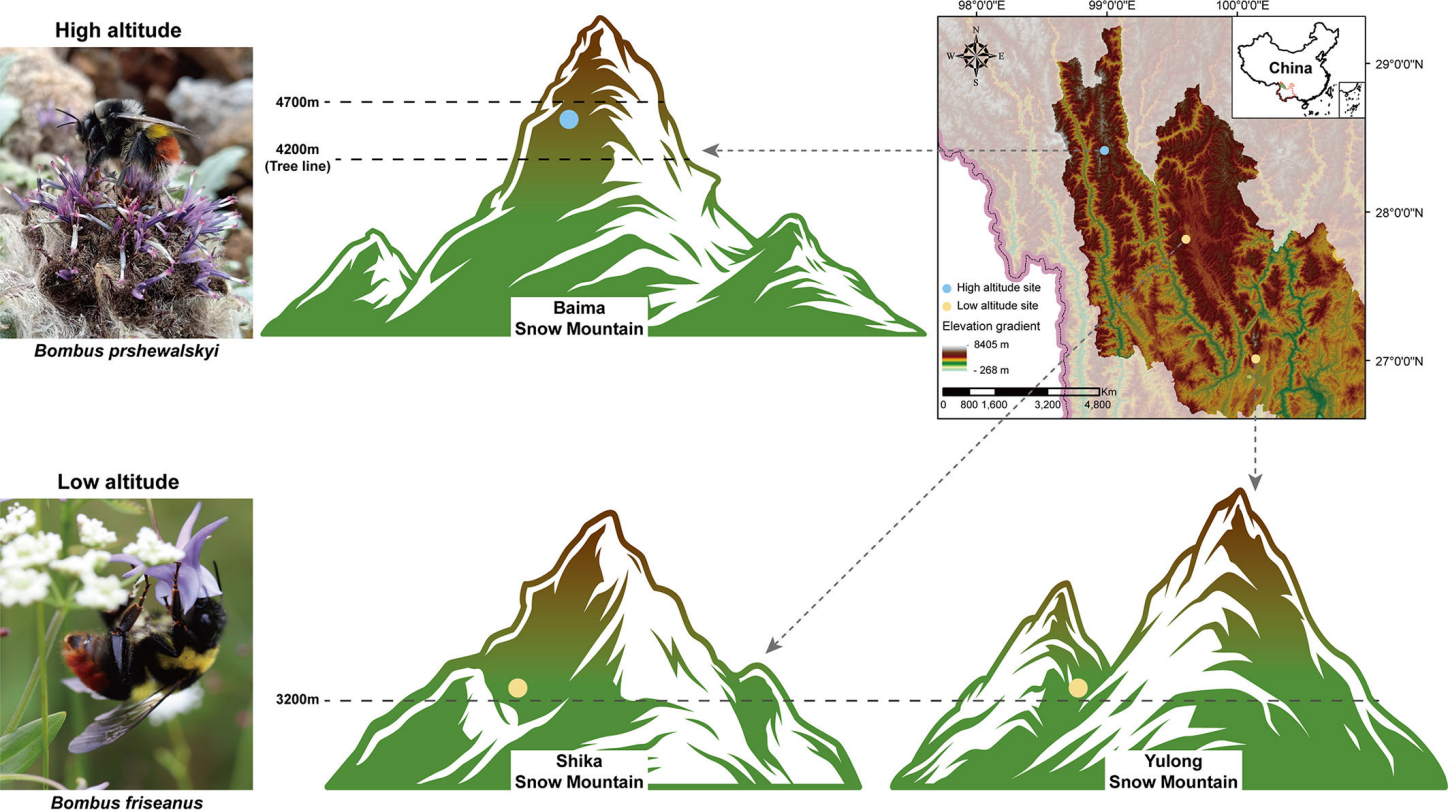
Sample sites of the two bumblebee species. Workers of *B. friseanus* were collected from the yellow dot sites on Yulong Snow Mountain and Shika Snow Mountain (3,200 m). Collection sites of *B. prshewalskyi* are marked with blue dots on Baima Snow Mountain above 4,200 m.

Deep-coverage metagenomic shotgun sequencing performed on all 20 samples for each species (Fig. 1; Table S1) resulted in an average of 36 Gbp for each sample. Subsequent processing divided each sample datum into two parts (Table S2). One part derived from the bumblebee host showed an average of 28.36 Gbp, and the second part derived from gut microbes had an average of 8.39 Gbp. Among 40 samples, 35 (*N* = 19 for *B. friseanus*; *N* = 16 for *B. prshewalskyi*) had over 15 Gbp corresponding to the host. The remaining five had 3.45–11.96 Gbp corresponding to the host. All 40 samples had over 1.2 Gbp corresponding to the gut microbiome.

### Host population structure and adaptation

**Fig 2:**
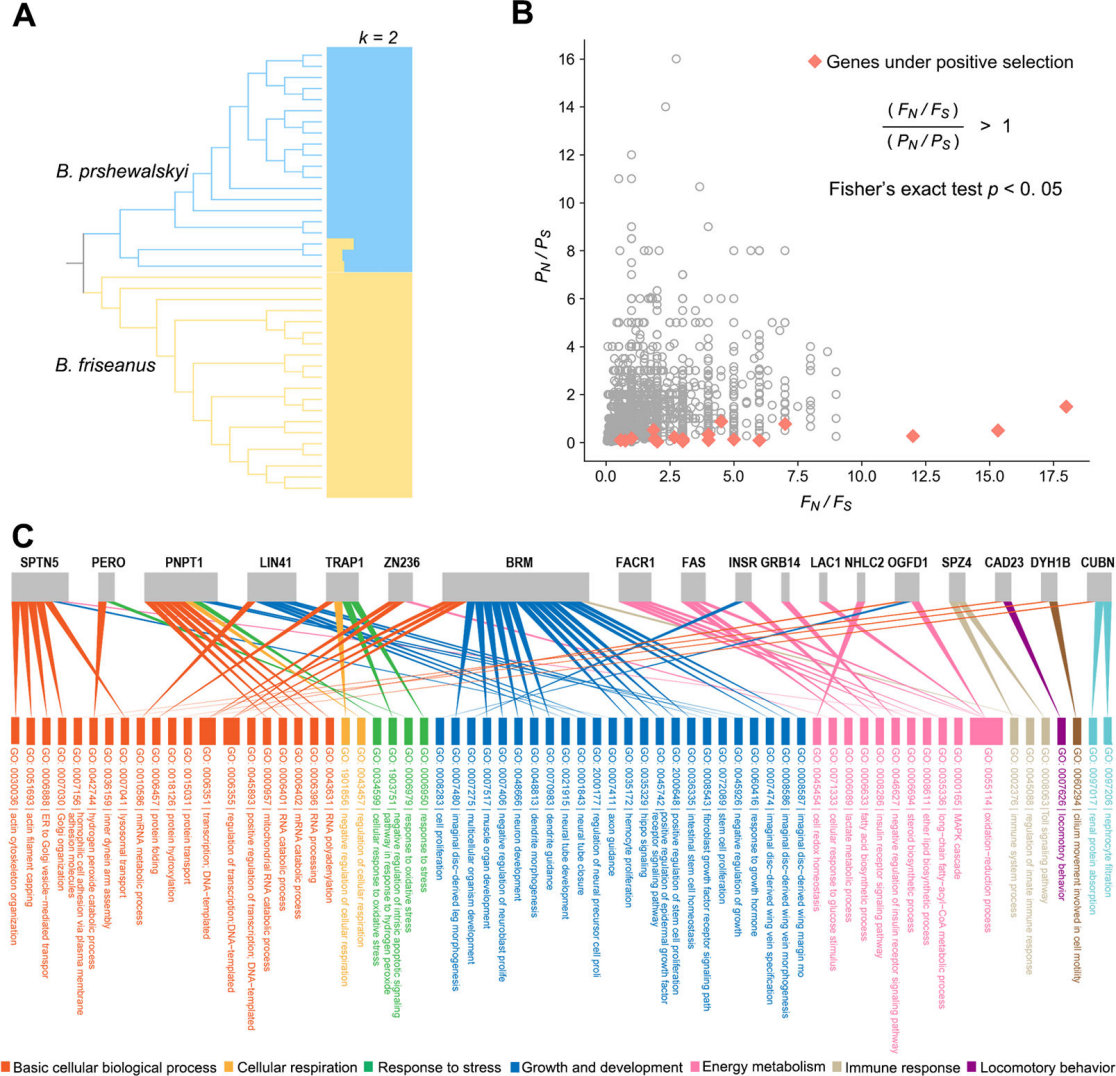
Host population differentiation and adaptation to higher (4,200 m) elevations. (**A**) Maximum likelihood tree and population structure of two bumblebee species. (**B**) The result of the McDonald–Kreitman test on coding genes of bumblebees. Orange diamonds indicate those genes under positive selection, while gray circles indicate other genes. *F_N_ / F_S_*: the fixed nonsynonymous/synonymous ratio between species; *P_N_ / P_S_*: the polymorphism nonsynonymous/synonymous ratio within species. (**C**) Candidate genes under positive selection are involved in diverse gene ontology (GO) processes. Lines indicate that genes are associated with the GO processes.

Genome alignment indicated an average of 112.5× sequencing coverage for each individual relative to a 225-Mb reference genome of *Bombus pyrosoma*. The average nucleotide identity (ANI) between *B. friseanus* and *B. prshewalskyi* genomes was 99.03%. A total of 112,132 single nucleotide polymorphisms (SNPs) were identified. Reduced genome-wide linkage disequilibrium and genetic diversity (π) were observed in the *B. prshewalskyi* populations at extremely high elevations (Fig. S1A and B). Based on linkage disequilibrium, the estimated effective population sizes of *B. friseanus* and *B. prshewalskyi* populations were 1,065 and 69, respectively. Population genetic analyses showed that *B. friseanus* and *B. prshewalskyi* populations formed two branches and divided distinctly in the maximum likelihood tree. This clear division was demonstrated further by genetic structure analysis at K = 2 (Fig. 2A). Principal component analysis (PCA) also supported the clear division of these two species. In PCA, the first principal component (PC) axes explained 11.18% of the total variation separating both bumblebee populations (Fig. S1C).

The McDonald–Kreitman (MK) test identified 23 candidate genes under positive selection from 2,009 genes with sufficient polymorphisms (Fig. 2B; Table S3). Eighteen of them were involved in multiple GO processes (Fig. 2C), ranging from basic to more complicated cellular processes. The basic cellular biological processes included the regulation of DNA transcription (GO:0006351, BRM, ZN236), RNA metabolic process [GO:0006396 (RNA), PNPT1; GO:0010586 (miRNA), LIN41], protein processing (GO:0015031, CUBN; GO:0006457, TRAP1; GO:0018126, OGFD1), actin cytoskeleton organization (GO:0030036, SPTN5), inner dynein arm assembly (GO:0036159, DYH1B), and hydrogen peroxide catabolic process (GO:0042744, PERO). The more complicated processes included response to stress/oxidative stress (GO:0006950, TRAP1; GO:0006979, PERO; GO:0034599, PNPT1), regulation of cellular respiration (GO:0043457, PNPT1; GO:1901856, TRAP1), developmental processes (GO:0035329, GO:0045742, BRM; GO:0008543, LIN41; GO:0045926, PNPT1), immune system processes (GO:0045088, BRM; GO:0008063, SPZ4), cell redox homeostasis (GO:0045454, NHLC2), cilium movement involved in cell motility (GO:0060294, DYH1B), locomotory behavior (GO:0007626, CAD23), oxidation–reduction processes (GO:0055114, FAS, FASC, OGFD1, LAC1), cellular response to glucose stimulus (GO:0071333, Zn236), and the insulin signaling pathway (GO: 0008286, INSR; GO: 0046627, GRB14). In general, all these functions were grouped within six physiological processes, namely, cellular respiration, stress response, growth and development, locomotory behavior, immune response, and energy metabolism.

### Microbiota composition and functions

**Fig 3:**
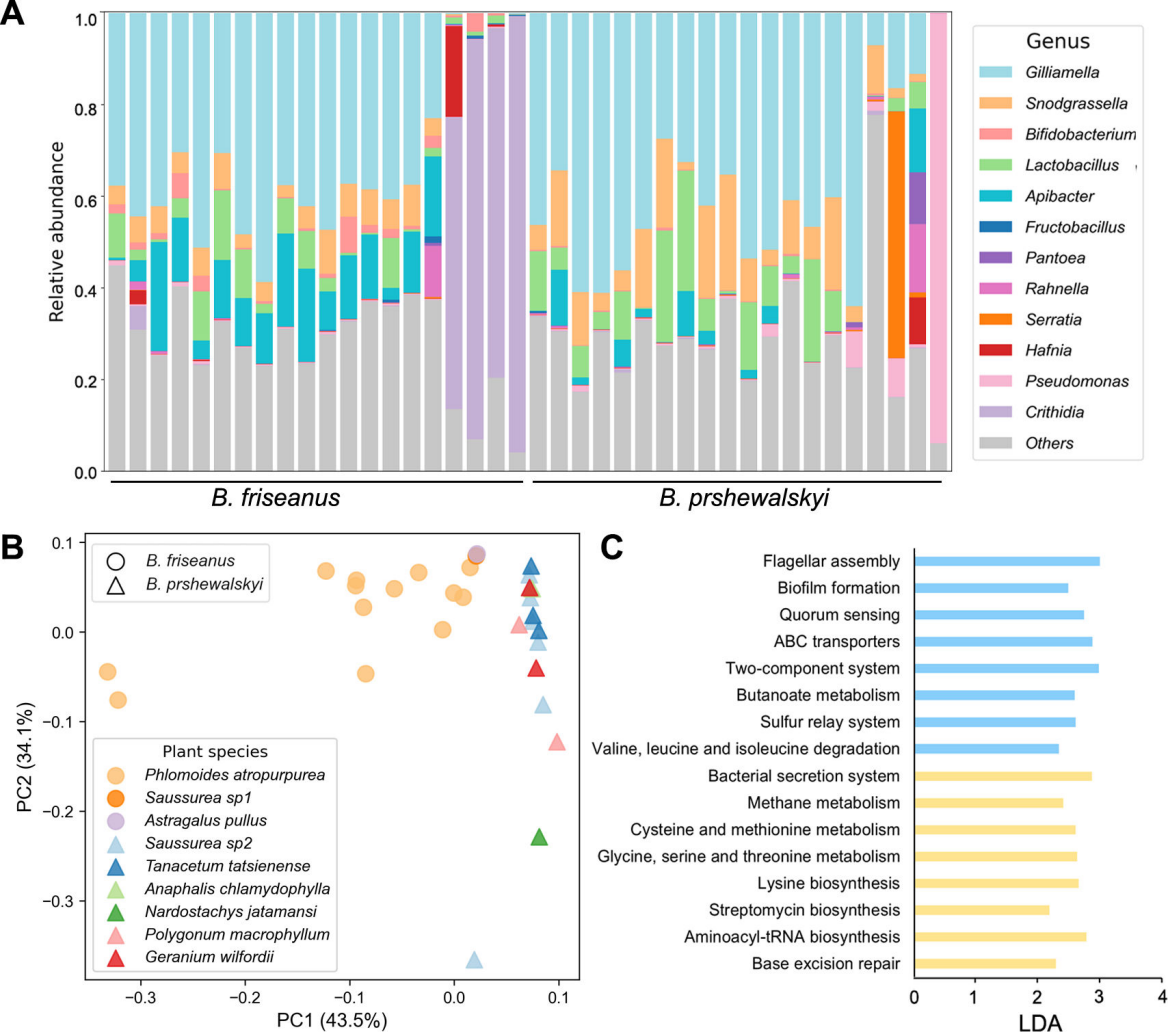
Gut communities of two bumblebee species differing in composition and function. (**A**) Taxonomic profiles of bumblebee gut microbiota estimated using the Kraken2 software. (**B**) Principal coordinate analysis (PCoA) based on the distance calculated by KmerFreqCalc. The clusters of bumblebee gut microbiotas were significantly associated with host specificity [permutational analysis of variance (PERMANOVA) *F*_1, 29_= 14.30, *r*^2^ = 0.33, *P* < 0.01] and related weakly with plant species foraged on by worker bees (PERMANOVA *F*_8, 22_= 3.07, *r*^2^ = 0.53, *P* < 0.05). (**C**) Kyoto Encyclopedia of Genes and Genomes pathways with differential relative abundances between gut microbiomes of *B. friseanus* and *B. prshewalskyi*.

Taxonomic profiles of bumblebee gut microbiomes estimated using the Kraken2 software showed that the core gut bacteria of bumblebees (i.e., *Gilliamella*, *Snodgrassella*, *Lactobacillus*, *Bifidobacterium*, and *Apibacter*) dominated most samples, although seven samples were dominated exclusively by pathogens. Specifically, four samples from *B. friseanus* were dominated by eukaryotic *Crithidia* sp., and three samples of *B. prshewalskyi* were dominated by potentially pathogenic bacteria including *Pseudomonas* sp.*, Serratia* sp.*, and Rahnella* sp. (Fig. 3A).

PCoA based on the distance calculated by KmerFreqCalc showed distinct clusters of the gut microbiomes from two bumblebee species. The first PC explained 43.5% and the second 34.1% (Fig. 3B). The two clusters were significantly associated with host specificity [permutational analysis of variance (PERMANOVA) *F*_1, 29_= 14.30, *r*^2^ = 0.33, *P* < 0.01). In addition, the difference between gut microbiomes showed a weak relation to the plant species foraged on by the worker castes of both species (PERMANOVA *F*_8, 22_= 3.07, *r*^2^ = 0.53, *P* < 0.05).

The Kyoto Encyclopedia of Genes and Genomes (KEGG) pathway enrichment analysis identified a total of 72 pathways in all samples. The LEfSe analysis showed pathways with significantly different abundances between the two bumblebee species involved in cell motility, cell community, membrane transport, signal transduction, amino acid metabolism, carbohydrate metabolism, and energy metabolism (Fig. 3C; Table S4). The relatively enriched KEGG pathways in the *B. prshewalskyi* gut microbiota included the flagellar assembly (ko02040), biofilm formation (ko02026), quorum sensing (ko02024), ATP-binding cassette (ABC) transporters (ko02010), two-component systems (ko02020), butanoate metabolism (ko00650), sulfur relay system (ko04122), and valine/leucine/isoleucine degradation (ko00280). In comparison, the more enriched pathways in the gut of *B. friseanus* included the bacteria secretion system (ko03070), methane metabolism (ko00680), lysine biosynthesis (ko00300), cysteine/methionine metabolism (ko00270), glycine/serine/threonine metabolism (ko00260), streptomycin biosynthesis (ko00521), aminoacyl-tRNA biosynthesis (ko00970), and base excision repair (ko03410).

### Metagenomic binning

**Fig 4:**
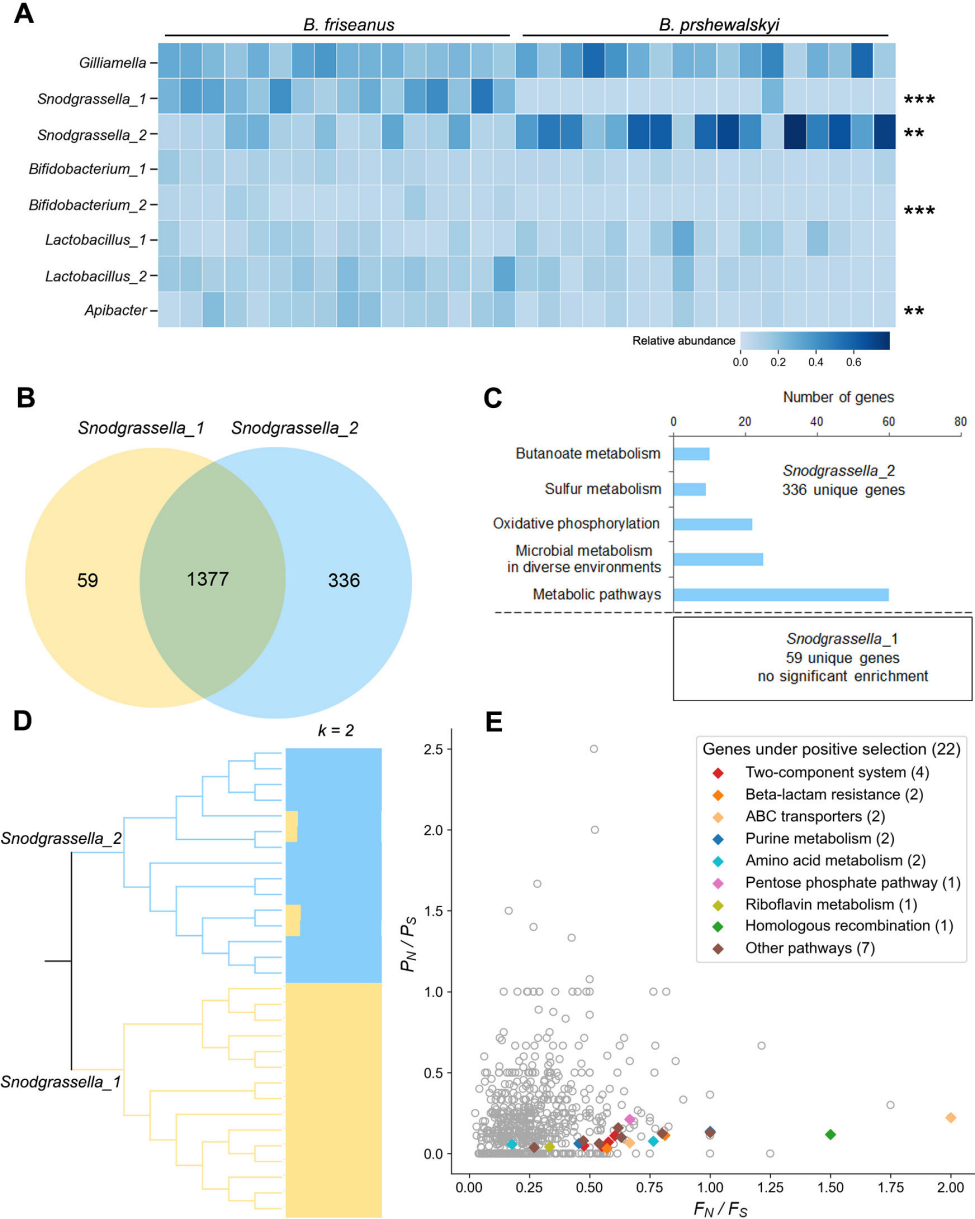
Metagenome binning revealed a pair of vicariously distributed species in *Snodgrassella* bacteria and the adaptation of *Snodgrassella* bacteria to the gut environment in a host restricted to a higher elevation. (**A**) The abundance of metagenome assembly bins in each healthy sample of bumblebees. The bins with significantly different abundances in two bumblebee species are marked with ** (*P* < 0.01, rank-sum test) or *** (*P* < 0.001, rank-sum test). (**B**) Comparison of gene compositions between two *Snodgrassella* bins using the genome of *Snodgrassella alvi*_wkB2 as the reference. (**C**) KEGG pathway enrichment analysis in the unique genes of *Snodgrassella* bins. (**D**) Maximum likelihood tree and population structure of two *Snodg rassella* bins. (**E**) The result of the McDonald–Kreitman test on genes in *Snodgrassella* bins. *F_N_ / F_S_*: fixed nonsynonymous/synonymous ratio between species; *P_N_ / P_S_*: polymorphism nonsynonymous/synonymous ratio within species. Colored diamonds indicate the genes under positive selection ((*F_N_ / F_S_*) / (*P_N_ / P_S_*) >1 and Fisher’s exact test *P* < 0.05) in diverse KEGG pathways, while gray circles indicate other genes.

Metagenomic binning yielded 23 bins showing >85% completeness and <5% contamination (Table S5). Taxonomic identification classified eight bins representing genera of eusocial bee gut core bacteria. This represented one bin from *Gilliamella*, two from *Snodgrassella*, two from *Lactobacillus*, two from *Bifidobacterium*, and one from *Apibacter*. Phylogenetic analysis showed that most of the core bacterial bins clustered together within typical strains of bumblebee gut bacteria, forming distinct clusters with their relatives in the honeybee gut, excluding the bin of *Snodgrassella*_1 located at the base of the *Snodgrassella* clade (Fig. S2). Quantitative analysis showed that the relative abundance patterns of four bins (*Snodgrassella*_1, *Snodgrassella*_2, *Bifidobacterium*_2, and *Lactobacillus*_2) differed significantly in the two bumblebee species (Wilcoxon rank-sum test, *P* < 0.01) (Fig. 4A; Fig. S3). Notably, *Snodgrassella* bins showed clear host specificity. *Snodgrassella*_1 dominated *B. friseanus*, while *Snodgrassella*_2 dominated *B. prshewalskyi*.

### Two *Snodgrassella* bins

The completeness of *Snodgrassella*_1 and *Snodgrassella*_2 bins was 94.77% and 88.82%, respectively. The ANI between the two bins was 77.65%. Phylogenomic analysis indicated that the two *Snodgrassella* bins differed significantly. *Snodgrassella*_1 clustered on the base of the *Snodgrassella* clade, while *Snodgrassella*_2 clustered with other strains from bumblebees (Fig. S2). Alignment with the genome of *Snodgrassella alvi*_wkB2 showed that each bin had unique genes (59 for *Snodgrassella*_1 and 336 for *Snodgrassella*_2), with 1,377 genes shared (Fig. 4B; Table S6). Five pathways showed enrichment in the 336 *Snodgrassella*_2 unique genes, including metabolic pathways, microbial metabolism in a diverse environment, butanoate metabolism, oxidative phosphorylation, and sulfur metabolism. No pathways were enriched in the 59 genes unique to *Snodgrassella*_1 (Fig. 4C). Further analysis indicated that genes involved in butanoate metabolism showed significant differences in the two bins (Fig. S4A). *Snodgrassella*_2 had twice as many genes involved in the butanoate synthesis pathway as *Snodgrassella*_1 (Fig. S4B).

To identify the adaptation signals of the two *Snodgrassella* bins, 15 sets of sequencing reads for each of the *Snodgrassella* bins were derived from raw data, identifying a total of 95,215 SNPs. Population genetic analyses, including population genetic structure (K = 2) and the maximum likelihood tree, showed a clear division between *Snodgrassella*_1 and *Snodgrassella*_2 (Fig. 4D). The MK test identified 22 candidate genes under positive selection from 496 with sufficient polymorphisms (Table S7; Fig. 4E). These genes involved in multiple KEGG pathways, including beta-lactam resistance (ko01501: two genes), two-component systems (ko02020: four genes), ABC transporters (ko02010: two genes), purine metabolism (ko00230: two genes), amino acid metabolism (ko00250: one gene, ko00260: one gene, and ko00400: one gene), riboflavin metabolism (ko00740: one gene), pentose phosphate pathway (ko00030: one gene), and homologous recombination (ko03440: one gene).

## DISCUSSION

We focused on two bumblebee species from the same subgenus as well as their gut microbiota, allowing us to hypothesize that genetic diversity in these two prospective host insects occurs along distinct zoogeographic ranges as shaped by adaptive selection. We also expected to detect differences between their gut microbiotas via metagenomic approaches, which may be involved in host genome differences.

We identified 23 genes under positive selection in *B. prshewalskyi* living at extremely high elevations. Consequently, it is not surprising that the gut microbiotas of both bumblebee species are significantly different in composition and function. Under positive selection, these 23 genes in *B. prshewalskyi* underlie diverse biological processes, implying that bumblebees adapt to extremely high elevations via complex and systematic mechanisms. Three genes associated with the regulation of cellular respiration (TRAP1 and PNPT1) and response to stress/oxidative stress (TRAP1, PNPT1, and PERO) significantly reflect the adaptation to hypoxia at greater heights. In particular, the positively selected BRM gene indicates that transcriptional regulation plays an important role in maintaining normal development and growth of bumblebees living under harsh conditions (i.e., lower ambient temperatures and hypoxia). A recent study on males of *B. terrestris* in Britain also showed that environmental pressures most likely contributed to recent changes in genes underlying physiology, neurology, and wing development (9).

Furthermore, BRM together with Spätzle (Spz) suggests ongoing stress on the bumblebees’ immune systems, with BRM regulating the innate immune response and SPZ in the Toll-like signaling pathway. The Toll-like signaling pathway is one of the most important intracellular signaling pathways in innate immunity systems in bees (24, 25) and other insects including *Drosophila* (26). In humans, acute high-elevation exposure upregulated the inflammatory signaling pathways and may have sensitized the toll-like receptor 4 (TLR4) signaling pathway to subsequent inflammatory stimuli (27). Further evaluation of the expression level and function of BRM or Spz and other immunological tests may provide additional support via future experimentation. Seven genes (FAS, FASC, OGFD1, LAC1, INSR, GRB14, and Zn236) involved in metabolic processes imply strong pressures on energy metabolism in montane bumblebees. Among them, INSR (insulin-like peptide receptor) and GRB14 are mentioned because they are involved in the insulin or insulin-like growth peptide signaling (IIS) pathway, an evolutionarily conservative nutrient-sensing pathway that modulates energy metabolism and development in metazoans (28, 29). In honeybees, the IIS pathway participates in the regulation of caste development (30–32) and the response to cold stress (33). The IIS pathway is also reported to contribute to the adaptive response to hypoxia in other insects including *Drosophila* (34, 35) and Tibetan locusts (6). Consequently, we propose here that the genetic variants of INSR and GRB14 in *B. prshewalskyi* play important roles in adapting bumblebee workers to hypoxia at extremely high elevations by regulating energy metabolism.

Additionally, the results suggest a potential correlation between the gut microbiota and those bumblebee adaptations, focusing on butanoate metabolism in the microbiota of *B. prshewalskyi* and the bins of *Snodgrassella*_2. Butanoate (or butyrate) is one of the short-chain fatty acids (SCFAs) generated by gut microbes. It can activate G-coupled-receptors directly, inhibit histone deacetylases, and serve as an energy substrate, regulating an animal’s physiology and health (36–38). In humans, butyrate is the primary energy source for colonocytes and also protects against colorectal cancer and inflammation (39, 40). It can improve the host’s metabolism of carbohydrates and lipids by serving as a signal molecule (41). Mice fed with a butyrate-enriched high-fat diet showed increased thermogenesis and energy expenditure and appeared resistant to obesity (42). Though simpler than the human or mice gut microbiota, the honeybee gut microbiota exhibits a variety of beneficial effects, from gut-centric functions like digestion (43), detoxification (44), and defense from pathogens (45), to more peripheral processes like behavior (46). The bumblebee gut microbiota has also been shown to provide some resistance to a trypanosomatid parasite (*Crithidia bombi*) (47). These effects are thought to be mediated, in part, by SCFAs (43). Considered synthetically, we propose that butyrate concentrations in the guts of bumblebees affect their energy metabolism and gut immunity. As both processes appear to be under stress at extremely high elevations, the results suggest that the gut microbiota may influence their hosts’ adaptations by adjusting the concentration of butyrate. Biochemical details of the impact effect of the gut microbiota on the bumblebee host should be elucidated further by experimentation on the gut butyrate and host physiology of these two bumblebee species or on related model species.

The different gut communities in the two bumblebee species reflect the hosts’ distinctive influence on their own gut microbiota. It is well known that host genetic background and diet are major factors influencing the gut microbiota (48–51). As plant species foraged upon by the bumblebee, samples were shown to correlate weakly with differences in gut microbiotas, and these differences may be mainly due to the hosts’ genetic background changes. The 22 genes involved in multiple KEGG pathways under positive selection in *Snodgrassella*_2 suggest strong host stress on their signaling pathway and antibiotic resistance process. *Snodgrassella* bacteria, forming a biofilm on the bumblebees’ ileum wall (15), directly encounter their host’s immune system. A recent study reported that the host specificity of honeybee gut bacteria was determined through reactive oxygen species that are regulated by immune deficiency and Toll pathways (52). Therefore, those host genetic variants involved in the immune system probably contribute to the species replacement of *Snodgrassella*.

In conclusion, we have helped to show that at extremely high elevations, *B. prshewalskyi* shows signs of positive selection in diverse biological processes, including cellular respiration, stress response, growth and development, locomotory behavior, immune system, and energy metabolism. We also found differences in both the composition and function of the bumblebee gut microbiome along an elevation gradient. Differences between the host bee species and their gut microbe species, as driven by adaptive selection, are probably functional and suggest a closer association between the genomes of hosts and their microbiomes. As adaptations shared by these bumblebee species with their microbiota indicate some degree of specialization, it may also reflect some degree of coevolution, but this will require further study.

## MATERIALS AND METHODS

### Bumblebee sampling

We collected workers of *B. friseanus* and *B. prshewalskyi* for gut extraction from 11 August to 15 August 2020. Workers of *B. friseanus* were collected between 2,700 and 3,200 m on Yulong Snow Mountain and the Shika Snow Mountain, northwest Yunnan. Collections of *B. prshewalskyi* came from 4,200 to 4,700 m on Baima Snow Mountain (Fig. 1, and Table S1 for the detailed information of sampling sites). As bumblebees are eusocial insects, we tried to avoid collecting individuals from the same colony by sampling more than one site on the three mountains for both species. The subsequent population genetic analyses of both species showed a high divergence among individual workers, suggesting that we did collect specimens from more than one colony (Fig. 2A).

We recorded the observation date, time, and plant species on which each foraging worker was collected. Each specimen was netted and stored in a separate 50 mL Eppendorf tube and euthanized at 4°C prior to gut dissection. Specimens were identified as species using morphological characters (18) under field conditions. Gut dissections and their preservation were performed in the field on the day of collection using a Stereo Microscope (Zeiss Stemi 508) as follows.

The surface of each bumblebee was first washed in droplets of 70% ethanol for 30 s and then rinsed with sterile water three times to remove external contaminants. We then pulled out the entire gut from the abdomen terminus using sterilized forceps. Each gut specimen was rinsed twice immediately with 0.9% sterile NaCl solution to maintain cell pressure. Each sample was stored separately in a 2mL cryotube placed in liquid nitrogen. After returning to the laboratory, gut specimens were frozen at –80°C until DNA extraction. In total, we collected 20 gut samples for each *Bombus* species.

### Metagenomic shotgun sequencing and processing

Total genomic DNA was extracted from gut materials using the E.Z.N.A. Soil DNA Kit (Omega Bio-Tek, Norcross, GA, USA). The concentration and purity of extracted DNA were determined with TBS-380 and NanoDrop2000, respectively. DNA extract quality was checked on a 1% agarose gel. The DNA extract was fragmented to an average size of about 400 bp using Covaris M220 (Gene Company Limited, China) for paired-end library construction. The paired-end library was constructed using NEXTFLEX Rapid DNA-Seq (Bioo Scientific, Austin, TX, USA). Adapters containing the full complement of sequencing primer hybridization sites were ligated to the blunt end of fragments. Paired-end sequencing was performed on Illumina NovaSeq 6000 (Illumina Inc., San Diego, CA, USA) at Majorbio Bio-Pharm Technology Co., Ltd. (Shanghai, China) using NovaSeq Reagent Kits, generating an average of 36 gigabyte bases.

Reads were first trimmed off with Trimmomatic v0.39 (parameters used: LEADING:3 TRAILING:3 SLIDINGWINDOW:4:15 MINLEN:40) (53) and inspected with FastQC v0.11.8 (54) to validate the trimming quality. Clean reads of each metagenomic sample were mapped against the reference genome of *B. pyrosoma* (GCA_14825855.1) using BWA v0.7.17-r1188 (55) to separate reads from the host and its microbes. *B. pyrosoma* belongs to the same subgenus as the two species, and its genome was annotated using Trinotate (http://trinotate.sourceforge.net/). About 80% of the total reads were derived from the host, and most of the remaining reads belonged to gut microbes. These two datasets were used in the subsequent bumblebee genomic and gut microbiome analyses.

### Population genetics and evolutionary analyses

The genome sequences of the two bumblebee species were generated based on the reference genome of *B. pyrosoma* and the alignment BAM files. The ANI between genomes was calculated using FastANI v.1.1 (56). To obtain high-quality SNPs, the BAM files were further processed as follows. PCR duplicates were removed by SAMtools v1.9 (57). The UnifiedGenotyper method in GATK v4.1.4.0 software (58) was used for SNP calling with default parameters across 40 individuals. To obtain a reliable SNP, we performed a filtering step with the following set of parameters: QD <2.0 || MQ <40.0 || FS >60.0 || SOR > 3.0 || MQRankSum <−12.5 || ReadPosRankSum <−8.0. The resulting VCF file was further filtered using VCFtools v 0.1.17 (59) (parameters: -remove-indels --max-alleles 2 --minDP 4 --minQ 70). We combined these methods to generate final SNPs. The effective population size (Ne) was estimated by NeEstimator (60) using the linkage disequilibrium method. Genome nucleotide diversity (π) was calculated using VCFtools v 0.1.17 (59). The significant difference between the two species was evaluated using the Wilcoxon rank-sum test. The linkage disequilibrium was calculated by PopLDdecay (61).

PCA for the first two components was performed based on the SNP site of the bumblebee in Plink (62). The resulting graph was plotted using the ggplot2 package in R (63).

The population structure was determined using ADMIXTURE v 1.3.0 (64). The estimation was K = 2 to K = 4, and the cross-validation (CV) error estimation was minimized at K = 2. A maximum-likelihood phylogenetic tree was constructed by the RAxML software (65), with the GTRGAMMA model and 100 bootstraps. An ascertainment bias correction was performed to correct for the impact of invariable sites in the data.

The MK test (8) was used to make inferences regarding historical selection by comparing fixed divergence and polymorphism at synonymous and nonsynonymous sites using a 2 × 2 contingency table. Significance was tested using Fisher’s exact test. To correct the false positives, the proportion of amino acid fixations driven by positive selection (α) was calculated following the procedure proposed by Shapiro et al. (66).

### Gut microbiome analyses

For each sample, the shotgun reads of the gut microbiome were randomly subsampled to reach the same sequencing depth using Seqtk v1.3-r106 (67) with parameters: -s 100, number = 1687456. Taxonomic profiles were estimated using the Kraken2 software (68) based on a custom comprehensive database, which was built with reference genomes of representatives of the Archaea, Eeubacteria, fungi, and protozoans. Relative abundance was estimated by Bracken (69). If *Crithidia* or pathogenic bacteria reads ever accounted for >50%, that sample was classified as “unhealthy individuals” and removed from downstream analyses. Similarity between samples was compared using KmerFreqCalc (*k* = 21) (70). PCoA was performed to illustrate the results. Significant differences between sample clusters across host specificity and diet type (the flower of the plant species on which a bumblebee foraged for nectar and/or pollen) were tested using PERMANOVA (adonis function in the vegan package v2.6-2, R software).

All host-filtered reads were used to build *de novo* assemblies separately using metaSPAdes v3.13.1 (71) with default parameters. The MetaGeneMark v3.38 (72) gene prediction tool was employed to predict genes, and functional profiling was determined by emapper.py from eggNOG mapper v2.1.3 (73). Gene abundance was quantified with Salmon v0.14.1 (74) using subsampled reads. KEGG pathway enrichment analyses were performed by ClusterProfiler v4.0 (75). The variance between annotated pathways was analyzed using the LEfSe software (76).

Metagenomic binning was performed following the metaWRAP pipeline (77). MetaSPAdes v3.13.1 (71) was used to produce co-assemblies for healthy samples from the same species. The completeness and contamination of each bin were estimated using CheckM v1.1.3 (78). Each bin was assigned taxonomically against the Genome Taxonomy Database with GTDB-tk v1.4.1 (79). Only bins meeting both criteria (completeness ≥80%; contamination ≤5%) were retained to calculate the ANI using FastANI v.1.1 (56). A 99% ANI threshold was set to distinguish strains from the same genus or family. For sibling bins, the one showing higher completeness and lower contamination was selected as one of the core bacterial strains in the bumblebee’s gut. Assemblies of core bacteria from the guts of other species of eusocial bees were downloaded from the NCBI to construct a phylogenetic tree with the bacterial bins using PhyloPhlAn v3.0.60 (80). Relative bin abundance was estimated by Salmon v0.14.1 (74). Rank-sum tests were used to determine whether the differences between bacteria abundance were significant.

### Comparative analysis of two *Snodgrassella* bins

Protein-coding regions were predicted using Prokka v1.14.6 (81). Predicted protein sequences were aligned to the genome of *Snodgrassella alvi* wkB2 (KEGG: T03063). Pathway enrichment analyses were performed with ClusterProfiler v4.0 (75). Genes involved in the butanoate metabolism pathway (ko00650) were compared between two *Snodgrassella* bins and highlighted in the KEGG pathway map.

Sequencing reads belonging to each bin were derived from metagenomic shotgun sequencing reads using BWA v0.7.17-r1188 (55) and SAMtools v1.9 (57). We used one of the *Snodgrassella* bins (*Snodgrassella*_2) as the reference genome to perform the subsequent admixture analysis and MK test as described previously.

## Data Availability

Whole metagenome shotgun sequencing datasets are deposited in the National Genomics Data Center (NGDC database) with the BioProject accession number PRJCA012598.

## SUPPLEMENTAL MATERIAL

Fig. S1-S4 msystems.01219-23-s0001.docx Genomic differences of two bumblebee species and the metagenomic analyses results.

Tables S1-S7 msystems.01219-23-s0002.xlsx Sample and sequencing dataset information, the results of population genetics and evolutionary analyses, and the results of metagenomic analyses.

ASM does not own the copyrights to Supplemental Material that may be linked to, or accessed through, an article. The authors have granted ASM a non-exclusive, world-wide license to publish the Supplemental Material files. Please contact the corresponding author directly for reuse.
