# Supplementary material for: The adaptation of bumblebees to extremely high elevation associated with their gut microbiota
Source: mSystems. 2024 Feb 8;9(3):e01219-23. doi: 10.1128/msystems.01219-23 (PMC10949452; doi:10.1128/msystems.01219-23)
Supplement: Fig. S1-S4 — Genomic differences of two bumblebee species and the metagenomic analyses results. [file msystems.01219-23-s0001.docx]

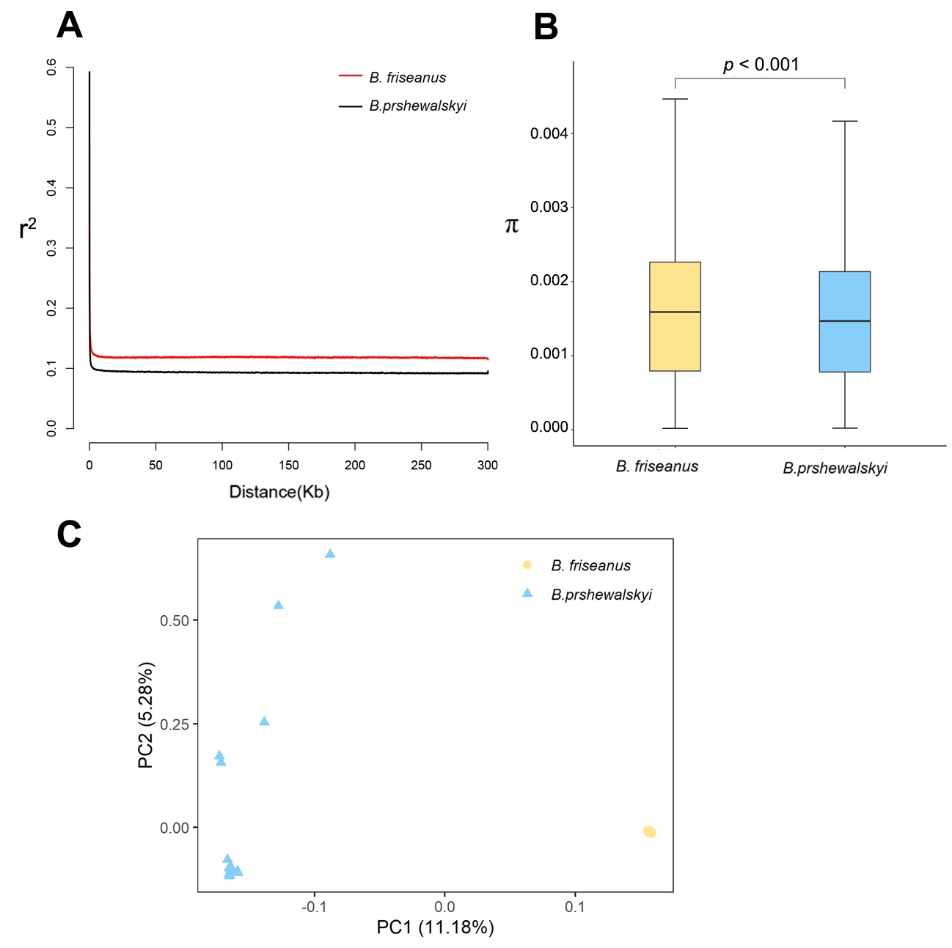


**FIG S1** Genomic difference between two bumblebee species. (A) Patterns of LD (linkage disequilibrium) decay across the genomes of two bumblebee species. r2, Pearson’s correlation coefficient. (B) Population nucleotide diversity (π) of two bumblebee species. Significant difference was evaluated using the Wilcoxon rank-sum test (*p* < 0.001). (C) PCA analysis of bumblebee genomes based on the SNP sites.


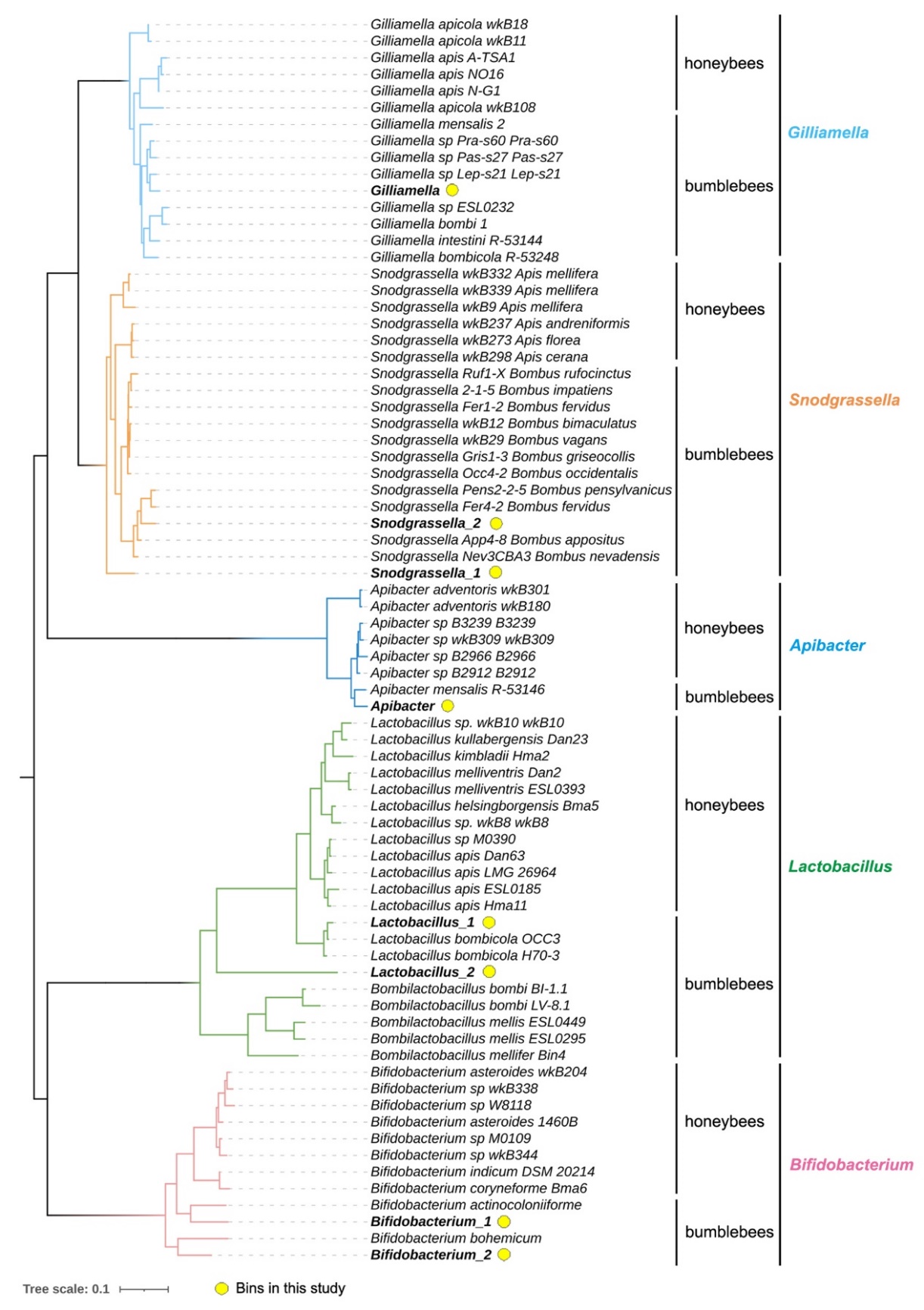


FIG S2 Maximum likelihood tree of our bins and previously reported genomes of social bee gut bacteria.

**
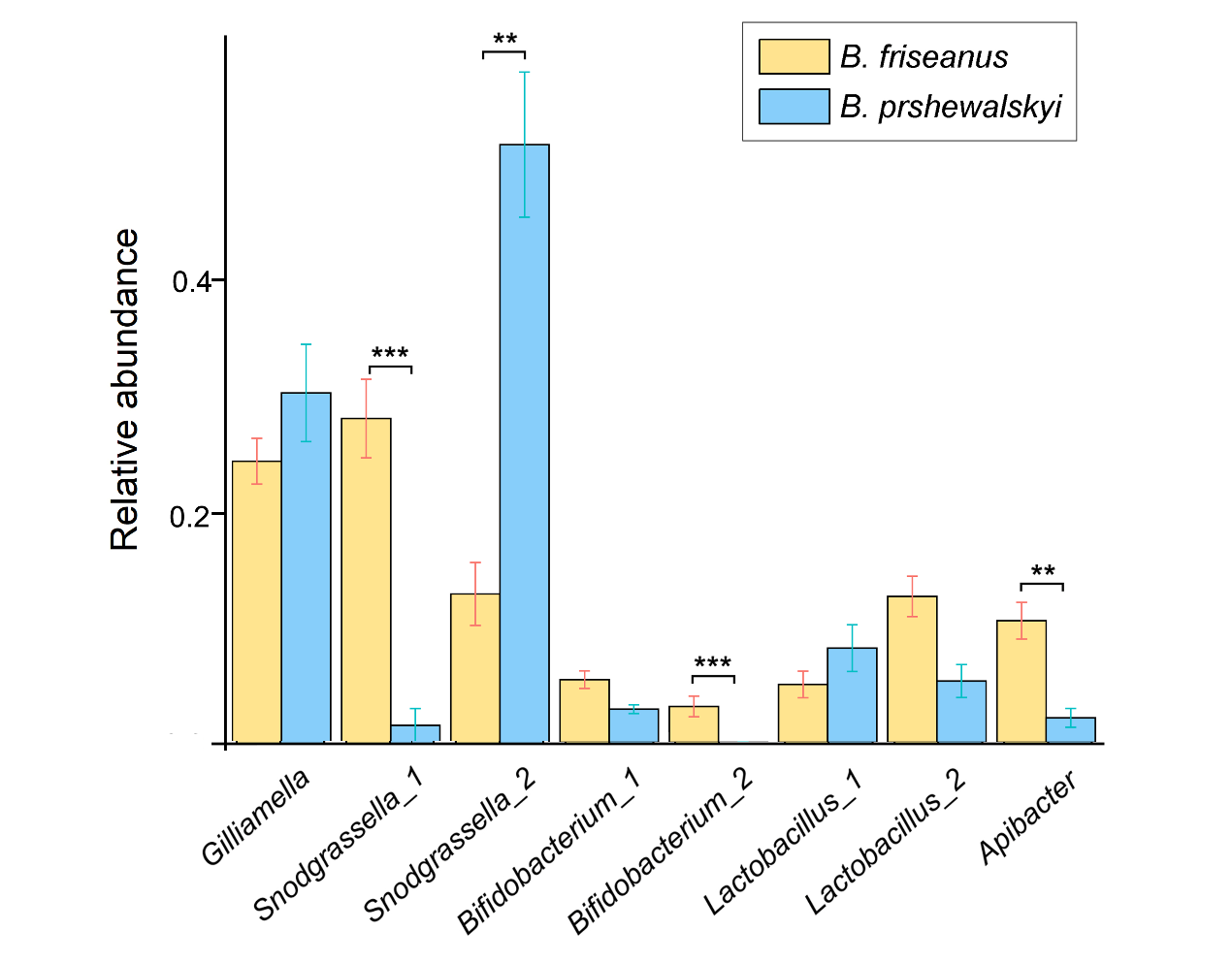
**

**FIG S3** The relative abundance of each bin in *B. friseanus* and *B. prshewalskyi*. Mean values ± standard errors of the means are shown. The bins with significant different abundance in two bumblebee species were mark with ** (*p* < 0.01, rank-sum test) or *** (*p* < 0.001).


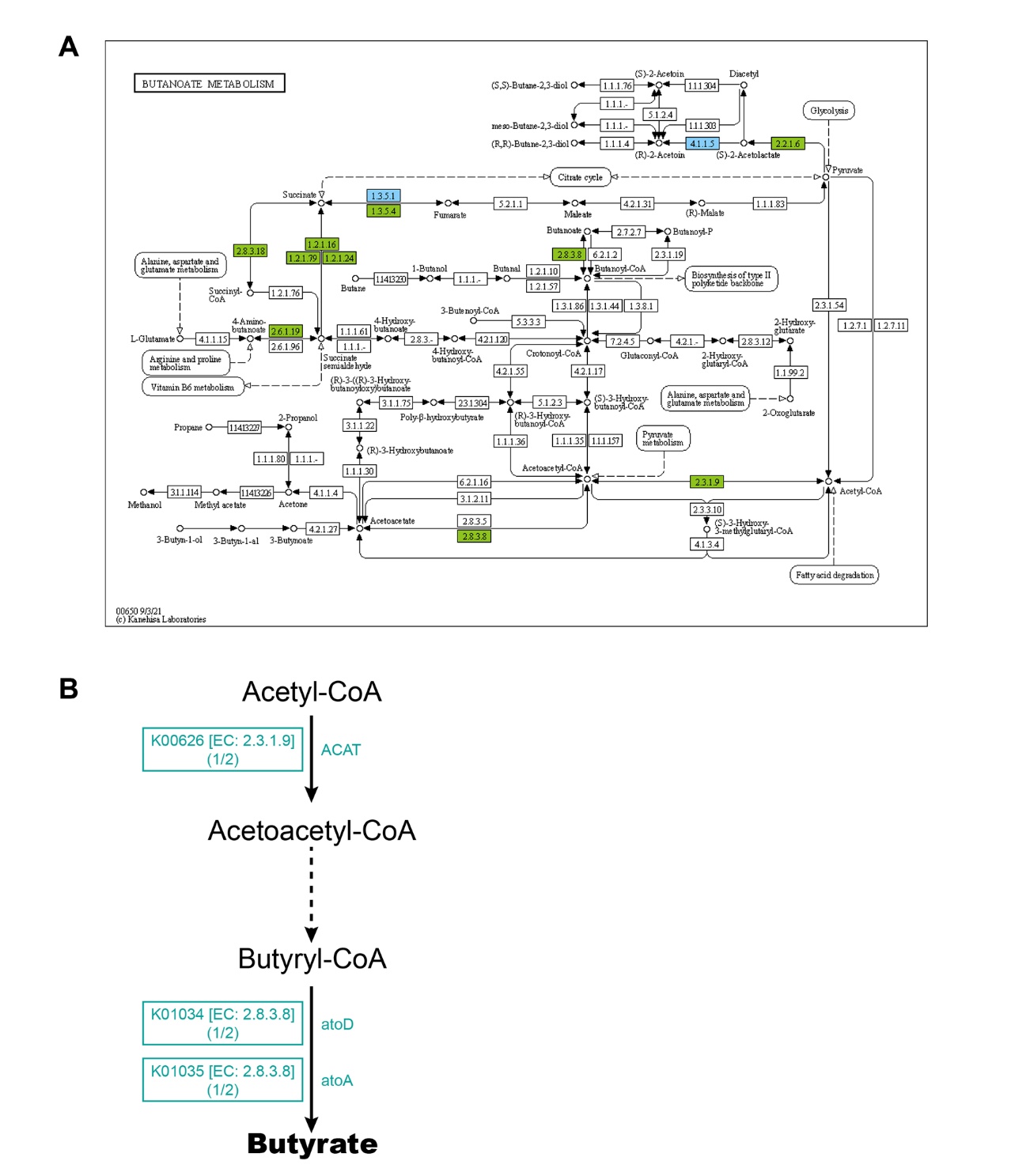


**FIG S4** Butyrate metabolism pathway in *Snodgrassella* bins. (A) Genes of two bins involved in butyrate metabolism pathway. Genes present in both two bins are indicated by green background. Genes only present in *Snodgrassella*_2 are indicated by blue background. (B) Illustration of the butyrate synthetic pathway. The number of genes in two bins (*Snodgrassella*_1/*Snodgrassella*_2) was labeled under the KO numbers.
